# Study on the Impact of Lithium Slag as an Alternative to Washed Sand on Mortar Properties

**DOI:** 10.3390/ma18153490

**Published:** 2025-07-25

**Authors:** Xianliang Zhou, Wei Dai, Xi Zhu, Xiaojun Zhou

**Affiliations:** 1 Key Laboratory of the Ministry of Education for Advanced Catalysis Materials, College of Chemistry and Materials, Zhejiang Normal University, Jinhua 321004, China; 2 Jinhua Xinsheng Zeolite Development Co., Ltd., Jinhua 321000, China; 3 School of Architecture and Civil Engineering, Xihua University, Chengdu 610039, China

**Keywords:** lithium slag, mortar, washed sand, macroscopic performance, microscopic analysis, chloride ion permeability resistance

## Abstract

Lithium slag (LS), a by-product of lithium extraction processes, poses a significant disposal challenge during the rapid development of new energy technologies. In this study, LS was used to replace partially washed sand in the process of mortar production to compensate for the content of stone powder in sand. Five mortar mixes containing varying proportions of LS were prepared, and the macroscopic performance was evaluated. A comprehensive microscopic analysis, including microstructure observations, hydration product identification, and pore structure analysis, was conducted. The impact of LS on the chloride ion permeability of mortar was also investigated in this study. The results indicate that an increase in LS content gradually reduces the workability of the mortar, with a 39.29% decrease in fluidity when 40% of the sand is replaced with LS. Moreover, the compressive and flexural strengths of the mortar initially increase and then decrease with higher LS content. Microscopic tests reveal that 20% LS substitution significantly optimizes the pore structure of the mortar, resulting in a lower chloride ion permeability coefficient. Consequently, 20% LS substitution is recommended as the optimal dosage for use as fine aggregate in mortar.

## 1. Introduction

As the world economy thrives and environmental awareness escalates, the development of the new energy industry is vigorously promoted worldwide. Lithium, a crucial component in various sectors, such as electric vehicles, aviation technology, and energy storage for electronic products, has triggered accelerated mining of lithium salts [1,2,3]. The global annual production of lithium salts exceeds 1.2 million tons, with China contributing over 800,000 tons [4]. However, under the current production process, approximately 10 tons of lithium slag (LS) waste is generated for every ton of lithium carbonate produced, posing a pressing challenge in proper disposal [5,6].

The prevalent methods for handling LS include landfilling or open-air storage, which not only consumes significant land resources but also raises environmental risks [7,8]. The sulfur content in LS can contaminate surrounding soil and groundwater through water diffusion. Therefore, the safe disposal of LS has become a significant focus of research [9,10]. A large number of scholars have begun to explore the application value of LS in the fields of ceramic materials [6], construction materials [11], biological electrode materials, and molecular sieves [12]. In addition, LS also has application prospects in silica–aluminum aerogels, glass ceramics, and other fields.

A wide range of industrial waste residues, including steel slag, high titanium slag, and phosphorous slag, are extensively utilized in construction materials [13,14,15]. This is due to the substantial demand for building materials, leading to the exhaustion of natural aggregates and irreversible environmental degradation [16,17]. Meanwhile, construction materials demonstrate remarkable versatility, allowing for the production of materials with varying properties from diverse raw materials [18,19]. For example, Santillán improved the electrical conductivity and mechanical properties of concrete by 70% and 14%, respectively, through the combined action of steel slag and metal fiber [20]. Liu prepared a series of new low-calcium CO_2_ sequestration cementitious materials by sintering the mixture of waste concrete fine powder and calcium carbide slag [21]. The combined application of industrial waste residues with concrete not only reduces the reliance on natural materials but also realizes the recycling of discarded resources, aligning with the principles of low-carbon and sustainable development. These research results provide a theoretical basis for the utilization of LS in the field of construction materials.

LS exhibits a similar particle size distribution to ordinary Portland cement and it is rich in silicon, aluminum, and calcium oxides, resulting in potential utilization in building materials [22,23,24]. Some researchers have denoted efforts to replace cement with LS in concrete, aiming to reduce the amount of cementitious material, thus reducing carbon emissions and costs [25,26,27]. However, the irregular shape of LS particles leads to a decrease in the workability of the paste as the LS content increases, particularly in terms of air content and slump, limiting its application [28,29]. Despite this, the compressive strength and durability of concrete can be enhanced when LS is used as a partial cement replacement. Rahman evaluated the pozzolanic activity of LS through various chemical tests and discovered that LS contains 31.6% amorphous phases, primarily composed of aluminosilicate phases with Na, Ca, and Mg, consistent with other commonly used pozzolans [30]. This indicates its potential as a pozzolanic material. However, regardless of whether LS is used as a cement replacement or a supplementary cementitious material, the actual utilization rate of LS remains relatively low compared to the huge quantities of available LS.

Most of the previous studies focused on the pozzolanic activity of LS, aiming to use it as an auxiliary gelling material. However, in addition to cementing materials in building materials, sand is also an essential component, even higher than the content of cementing materials. Washed sand is widely used in engineering. However, due to the addition of flocculant to the washed sand, the partial stone powder and the mud in the sand are washed together. The lower content of stone powder is unfavorable to the working performance of concrete, such as the reduction in wrapping properties. Replacing part of washed sand with LS can increase the content of stone powder in the sand and then change the grading of the sand. However, little research has been reported in this area. In a small number of similar studies, it is not clear how LS as a substitute for sand affects the properties of building materials. The high SiO2 content in LS, coupled with its microscopic morphology resembling that of manufactured sand, presents a novel utilization path as a potential sand substitute, meriting further exploration [31]. Dong utilized LS as an ultrafine aggregate at different sand replacement levels in the preparation of mortar. The results indicate that LS can significantly reduce the structural deadweight and enhance the flexural and compressive strength and peak stress of the mortar [32,33]. Moreover, the utilization rate of LS as an ultrafine aggregate is five times higher than when it is used as a supplementary cementitious material. As an emerging industrial waste residue, LS has a relatively limited distribution, leading to processing challenges. The research on LS as a fine aggregate substitute remains insufficient and needs investigation. Previous studies have primarily focused on macroscopic-level observations, lacking in-depth analysis of the mechanisms governing how LS impacts the performance of mortar or concrete as a fine aggregate. This research is crucial for enhancing the resource utilization rate of LS and promoting its wider application.

This study is committed to investigating the impact of using LS as a substitute for fine aggregate on the macro-properties and micro-characteristics of mortar. By employing various replacement ratios (10%, 20%, 30%, 40%), LS is substituted for manufactured sand by weight. Firstly, this study explored the influence of different LS replacement ratios on macro-properties, such as mortar fluidity, compressive strength, and flexural strength. Subsequently, microscopic analysis was conducted to understand the mechanisms underlying the effects of LS on mortar, including microstructure, hydration products, hardened pore structure, and chloride ion permeability. The primary objective of this research is to determine the optimal replacement rate of LS as a substitute for fine aggregate in mortar. This study can provide a comprehensive understanding of the feasibility, optimal replacement rate, and mechanism of action of LS as a fine aggregate substitute in mortar, thus laying a solid theoretical foundation for conserving natural sand and broadening the application of LS.

## 2. Experimental Investigation

### 2.1. Raw Materials

**Figure 1:**
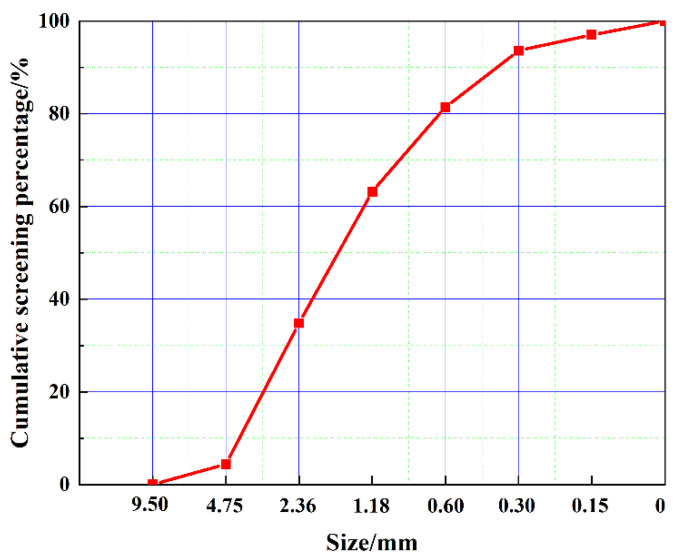
Sand grading curve.

**Figure 2:**
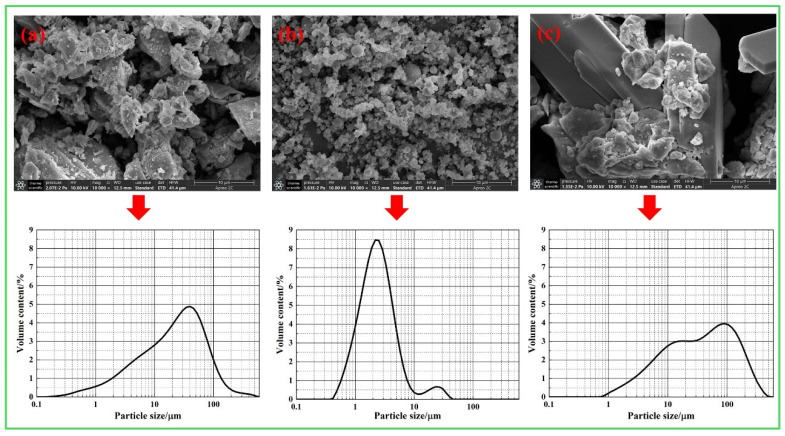
Microstructures and particle size distribution curves of the raw materials: (**a**) cement; (**b**) fly ash; (**c**) LS.

The cement utilized in the experiments is class 42.5 Ordinary Portland cement manufactured by the China Conch Group, and the 3-day and 28-day compressive strengths are measured at 23.7 MPa and 47.6 MPa, respectively, in accordance with the GB 175-2023 [34] standard. The fly ash (FA) was sourced from Donglanxing New Materials Co., Ltd., in Chengdu, Sichuan Province. The discarded LS, which originated from the lithium salt plant in Mianyang, Sichuan Province, exhibits a natural water absorption rate of 22.6%. The primary chemical compositions of the cement, FA, and LS are presented in Table 1. The sand used in the experimental tests is manufactured sand with a fineness modulus of 3.64. The grading curve of sand is shown in Figure 1. It can be seen that the particle size of the sand used in the test is larger, which is to better highlight the grading adjustment effect of LS on sand. Figure 2 illustrates the microstructure and particle size distribution of the cement, FA, and LS. It can be seen that the particle size of LS is the largest, which is very close to the particle size of stone powder in sand.

### 2.2. Mix Proportions

Using cement and fly ash as binder materials and manufactured sand as a fine aggregate, a control group was developed and named SR0 for the preparation of mortar. Subsequently, four experimental groups with different LS contents were prepared to replace manufactured sand by weight. Based on the replacement levels, these groups were designated as SR10, SR20, SR30, and SR40. All five groups of the samples were cast with a water-to-binder ratio of 0.3. The workability of the mortar was adjusted using a superplasticizer. The polycarboxylic acid superplasticizer used in the experiment was provided by Xiamen Jibang New Materials Co., Ltd. (Xiamen, China), with a water reduction rate of 25%. Due to the larger specific surface area and water absorption rate of LS compared to manufactured sand, an increased dosage of superplasticizer was required to maintain a similar workability to the control group. The purpose is to avoid any impact on the performance of the mortar due to differences in fluidity. The mix design for five groups of samples is presented in Table 2.

### 2.3. Sample Preparation

**Figure 3:**
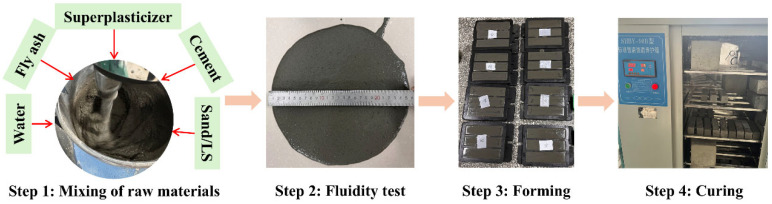
Specimen preparation and curing process.

The preparation process for the mortar is outlined as follows: Firstly, the cement, FA, manufactured sand, and LS were placed in a mortar mixer and mixed for 1 min to ensure uniform distributions. Subsequently, water and superplasticizer were gradually added to the mixture during the mixing process. Once the addition of water and superplasticizer was complete, the mixture was further mixed for 2 min. After mixing, the mortar was subjected to a fluidity test. The mixture, following the fluidity test, was then poured into molds of varying sizes for molding, involving vibration to ensure the uniformity of the samples. To prevent water evaporation from the mortar, the top of the mold was covered with a film. After maintaining the samples in the mold for 24 h, they were removed and placed in a standard curing chamber (a temperature condition of 20 ± 2 °C and relative humidity condition of 95%). Figure 3 illustrates the sample preparation process. Prismatic specimens of 40 mm × 40 mm × 160 mm were used for compressive and flexural strength testing. Cubic specimens of 100 mm × 100 mm × 100 mm were used to characterize the pore structure of the samples. Cylindrical specimens with a diameter of 100 mm and a height of 50 mm were utilized for the chloride ion permeability test.

### 2.4. Test Procedures

#### 2.4.1. Fluidity Test

The fluidity test of the mortar was conducted in accordance with Chinese Standard GB/T 2419-2005 [35]. To ensure the accuracy of the tests, the fluidity of each sample was measured three times. To investigate the impact of LS content on mortar fluidity, the same amount of superplasticizer as in the control group (SR0) was added to the mortars with varying LS substitution rates. Considering the fluidity of the control group as a reference, the fluidity of the mortars with different LS substitution rates was adjusted to a similar level by increasing the dosage of superplasticizer.

#### 2.4.2. Compressive and Flexural Strength Test

The compressive and flexural strength tests for the mortar were performed in accordance with the “Test Method of Cement Mortar Strength” (GB/T 17671-2021 [36]). These tests are conducted at 3-day, 7-day, and 28-day curing ages to measure the compressive and flexural strengths of the mortar. The flexural and compressive strengths of each mortar group were determined as the average of 3 and 6 samples, respectively.

#### 2.4.3. Scanning Electron Microscope (SEM) Test

The microstructures of mortar with varying LS substitution rates were observed using a Scanning Electron Microscope (SEM). During the test, the equipment voltage remained at 5 kV, and the microscopic features of the sample were observed through different amplification factors. The test samples are intact fragments remaining after the compressive strength testing, ensuring minimal disturbance to their original structures. The test samples were dried in an oven at a temperature of 108 °C.

#### 2.4.4. X-Ray Diffraction (XRD) Test

The test results for indicators such as the type and quantity of hydration products can provide a microscopic perspective on the influence of LS on the mechanical properties of mortar. Since sand does not affect the hydration process of cement but may introduce substances, like silica, that could compromise the accuracy of hydration product testing, the samples in Table 1 were re-prepared without sand for hydration product testing. After 28 days of curing, the prepared samples were dried and grinded. X-ray diffraction (XRD) analysis was then conducted to determine the type and content of crystalline phases in the samples with different LS contents. The mineral composition of the mortar was analyzed using the DX-2800 X-ray diffraction instrument produced by China Dandong Haoyuan Instrument Co., Ltd. (Dandong, China).

#### 2.4.5. Pore Structures

**Figure 4:**
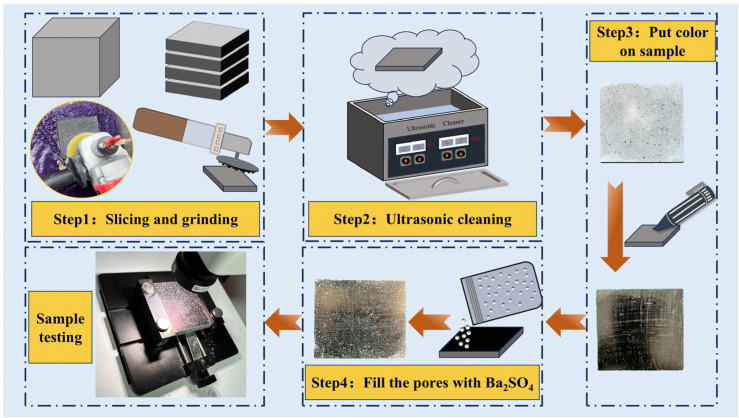
Test procedure for pore structure analysis of mortar.

Cubic specimens of 100 mm × 100 mm × 100 mm were prepared to investigate the pore structure of mortar under different LS substitution rates. After 28 days of curing, the specimens were sliced into 15 mm × 100 mm × 100 mm samples using a cutting machine. For each test piece, three central slices were selected for further experimentation. These slices were then grinded using silicon carbide abrasive papers of varying fineness (50, 100, 300, 800, 1500, and 3000 grit) with the assistance of a grinding machine. Each abrasive paper was used for 3 min of grinding. The slices were then cleaned using an ultrasonic cleaner. The cleaned samples were coated with a black marker to darken their surfaces and enhance comparison by filling them with 20 nm white barium sulfate powder. Finally, the samples were scanned using a hardened concrete pore structure analyzer (HC-457), which is produced by Hangzhou Guanli Intelligent Technology Co., Ltd. (Hangzhou, China). Figure 4 illustrates the detailed testing procedure.

#### 2.4.6. Chloride Ion Permeability Resistance Test

Drawing inspiration from Chinese Standard GB/T 50082-2009 [37], this study explores the impact of LS substitution for varying sand types on the chloride ion penetration resistance of mortar. After the completion of the test, the chloride ion penetration depth at 42 points was measured on the sample, and the average penetration depth was calculated. The chloride ion permeability coefficient of different mortars was calculated using Equation (1):

(1) $$D_{RCM}=\frac{0.0239\times (273+T)L}{(U-2)t}\left(\left.X_{d}-0.0238\sqrt{\frac{(273+T)LX_{d}}{U-2}}\right)\right.$$

where $D_{RCM}$ is the unsteady-state chloride ion migration coefficient, expressed with a precision of 0.1 × 10^−12^ m^2^/s; U is the absolute value of the applied voltage (V); T is the average of the initial and final temperatures of the anode solution (°C); L is the thickness of the specimen (mm), expressed with a precision of 0.1 mm; $X_{d}$ is the average chloride ion penetration depth (mm), expressed with a precision of 0.1 mm; and t is the duration of the test (h).

## 3. Experimental Results and Discussion

### 3.1. Workability

**Figure 5:**
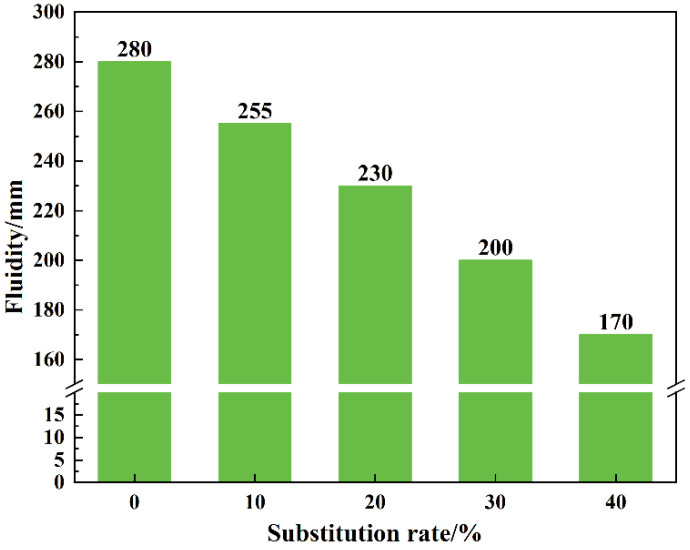
The impact of lithium slag dosage on the workability of mortar.

The influence of LS content on mortar workability can be better represented without altering the dosage of superplasticizer, as presented in Figure 5. An increase in the proportion of LS substitution in the mortar results in a significant decrease in its workability. As the substitution rate rises from 0% to 40%, the workability drops from 280 mm to 170 mm, representing a reduction of 39.29%. This phenomenon is attributed to the finer particle size of LS, leading to a larger specific surface area compared to sand, enabling it to adsorb more water. Additionally, LS exhibits a higher water absorption capacity. These two factors contribute to a decrease in free water content and an increase in frictional forces within the mortar, ultimately reducing its workability. It is noteworthy that the addition of a small amount of superplasticizer can mitigate this issue, as evident from Table 1, where varying admixture dosages enable each mortar mix to achieve similar workability.

### 3.2. Mechanical Properties

**Figure 6:**
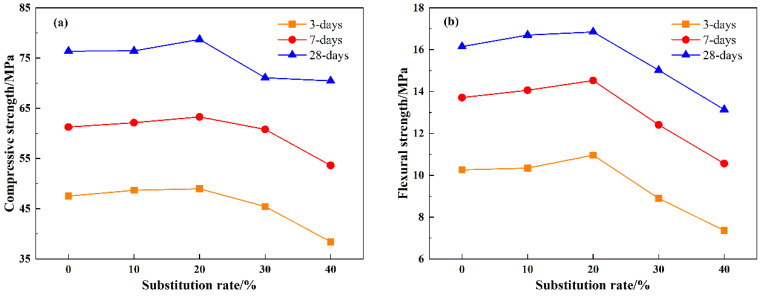
The impact of different slag admixtures on the compressive strength and flexural strength of mortar at different ages: (**a**) compressive strength, (**b**) flexural strength.

Figure 6 depicts the compressive and flexural strengths of mortar at 3 d, 7 d, and 28 d curing ages with varying LS dosages. It is evident from Figure 6 that the trend in compressive and flexural strengths for all three curing ages follows a similar pattern: an initial increase followed by a decrease. Specifically, mortar with an LS dosage of 20% exhibits the most superior mechanical properties. This is attributed to the fine-grained LS effectively filling the internal pores of the mortar. However, as the dosage exceeds 30%, the strength of the mortar begins to decline, falling below the control group. This observation can be explained by three main factors: Firstly, with a high binder content and fewer internal pores, there is less LS available for pore filling. Secondly, the remaining lithium slag functions as aggregate, but its strength is significantly lower than that of natural fine aggregates. Thirdly, the reduced content of natural fine aggregates provides support and contributes to the decline. Furthermore, when compared to the control group, the compressive strengths of the mortar with a dosage of 40% at 3 d, 7 d, and 28 d decreased by 19.17%, 12.47%, and 7.74%, respectively. Similarly, the flexural strengths of SR40 at 3 d, 7 d, and 28 d are reduced by 28.27%, 22.98%, and 18.64% compared to SR0. This phenomenon indicates that high LS dosages primarily impact the early-age strength of mortar because LS slows down the early hydration reactions of cement.

### 3.3. Microstructure Analysis

**Figure 7:**
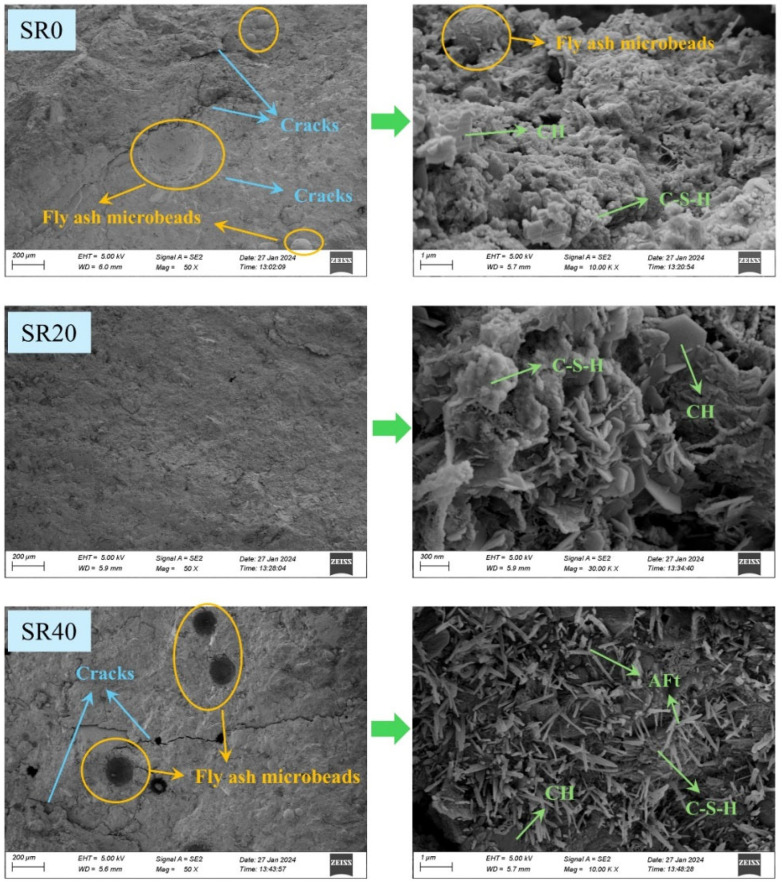
Microstructures of mortar with different LS content.

A comparative analysis of the microstructure of mortars with 0%, 20%, and 40% lithium slag content is presented in Figure 7. Mortar without LS exhibited a relatively dense internal structure, containing only a minimal amount of cracks. Additionally, it demonstrated a high degree of hydration, where most calcium hydroxide (CH) transformed into hydration products, such as calcium silicate hydrate (C-S-H). Upon the substitution of 20% sand with LS, the microstructure became the most compact, practically devoid of cracks. The degree of hydration remained high within the mortar. However, with a 40% LS mixture, the microstructural density of the mortar significantly decreased, resulting in the appearance of cracks and even voids. Moreover, the surface of the hydration products exhibited a substantial amount of needle-like ettringite (AFt) crystals. Previous studies have indicated that the introduction of substances with high SO_3_ content into cement paste promotes the formation of AFt [38,39]. As AFt is a crystal with extremely high water content, its formation consumes a significant amount of free water, thereby reducing the amount of water available for cement hydration and subsequently diminishing the quantity of hydration products. Furthermore, the expansive nature of AFt also contributes to the reduction in mortar structural density, leading to an increase in cracks and voids [40,41].

### 3.4. Hydration Products

**Figure 8:**
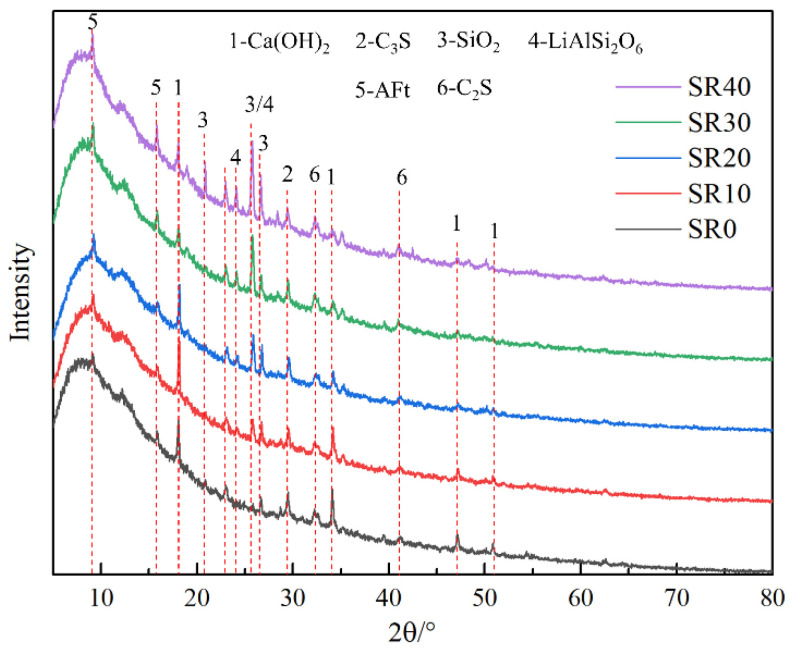
Analysis of crystal components in mortar with different LS content.

The composition of mortar with different lithium slag content was analyzed using XRD testing, as shown in Figure 8. The crystalline components in the five groups of mortars primarily comprised unreacted or insufficiently reacted silica (SiO_2_), dicalcium silicate (C_2_S), tricalcium silicate (C_3_S), and calcium hydroxide (CH) formed after hydration. As the LS content increased, the diffraction peaks of calcium hydroxide in the mortar gradually weakened, indicating a decrease in the quantity of calcium hydroxide and a lower degree of hydration within the mortar. This explains the reduction in mortar strength and the decreased compactness of its microstructure. Additionally, the diffraction peaks of spodumene (LiAlSi_2_O_6_) and gypsum (CaSO_4_∙2H_2_O) became more prominent as the LS increased. This phenomenon suggests that the presence of SO_3_ in the LS leads to the formation of new substances. According to previous research, a small amount of SO_3_ can have a positive impact on mortar performance, while an excessive amount can have a negative impact [9]. This is a significant reason why the mortar performance rapidly deteriorated when the LS exceeded 30% due to high SO_3_ content.

### 3.5. Air Content Properties

**Figure 9:**
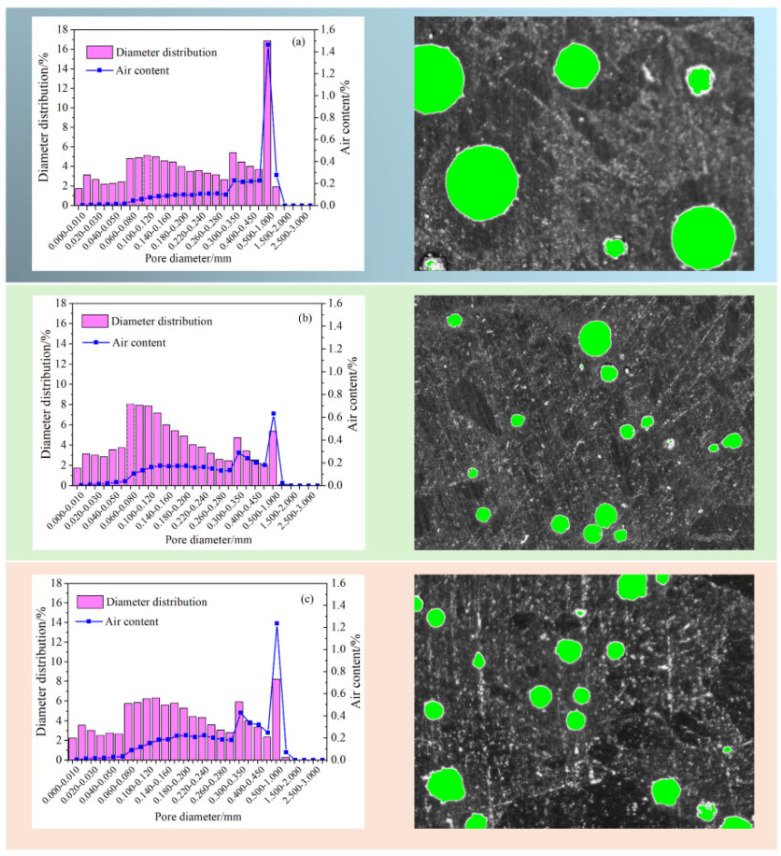
Air content and the spacing factor of hardened mortar. (**a**) SR0; (**b**) SR20; (**c**) SR40.

An analysis of the air content properties of three mortar mixtures, SR0, SR20, and SR40, after 28 days of curing is presented in Figure 9. The results reveal that the air content in the mortar initially decreased and then increased with an increasing amount of LS. Specifically, the air contents of SR0, SR20, and SR40 were 3.74%, 3.51%, and 4.92%, respectively. A moderate amount of LS as a sand substitute can reduce the internal air content of the mortar. However, an excessive amount of LS leads to an increase in internal air content. Two main reasons account for this phenomenon. Firstly, a high LS admixture can affect the hydration of cement, reducing the compactness of the mortar. Secondly, as the internal relative humidity decreases, LS with high water absorption releases water, leading to the formation of pores. Many industrial wastes with similar physical and chemical properties to LS exhibit the same phenomenon when they are used as substitutes for sand [42,43,44].

Similar to the air content, the air bubble spacing factor exhibited a trend of first decreasing and then increasing with an increasing lithium slag admixture. However, interestingly, SR40 exhibited a smaller bubble spacing compared to SR0. This is attributed to the addition of LS, reducing the number of large air bubbles and increasing the number of small air bubbles in the mortar [45,46].

**Figure 10:**
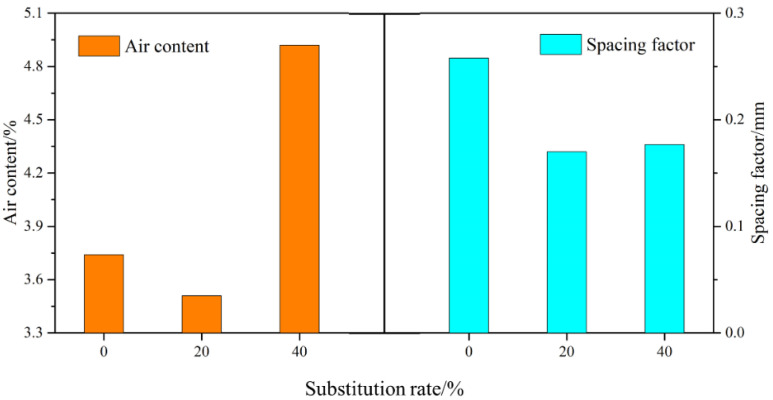
Pore diameter distribution, air content, and pore distribution characteristics of mortar.

The distribution of pore diameters, air content, and pore distribution characteristics in mortar are illustrated in Figure 10. With variations in the LS replacement, the proportion of pores with different diameters within the mortar underwent significant changes. Overall, the addition of lithium slag noticeably reduced the proportion of large-diameter pores and increased the proportion of small-diameter pores, owing to the filling effect of LS. However, this modification effect tended to weaken as the LS increased. The pore distribution characteristics further validate these observations. In SR0, the number of pores was small, but their diameters were significant. The incorporation of LS reduced the pore diameters but increased the number of pores. The increase in large-diameter pores in SR40 is attributed to the impact of LS on the workability.

### 3.6. Chloride Ion Permeability Resistance

**Figure 11:**
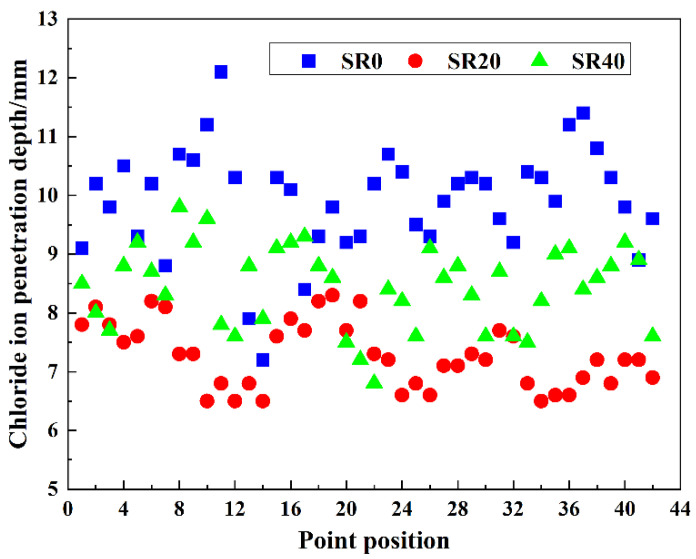
Chloride ion penetration depth at different points of mortar with different LS content.

**Figure 12:**
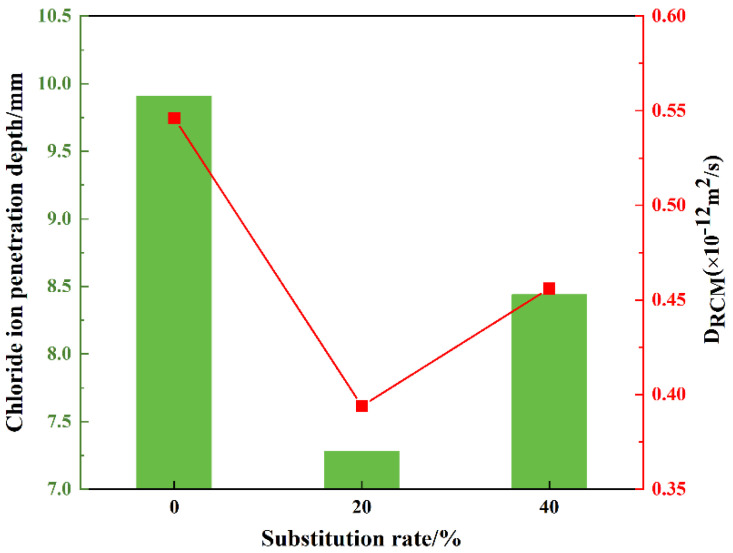
The average chloride ion penetration depth and chloride ion permeability coefficient of mortar with different LS content.

The chloride ion penetration depths at 42 distinct locations on three mortar mixtures, including SR0, SR20, and SR40, were measured, as shown in Figure 11. The average penetration depths and permeability coefficients were calculated, as presented in Figure 12. Evidently, the overall trend in chloride ion penetration depths across different locations in the three mortar mixtures was consistent, indicating the scientific agreement of the results. An appropriate amount of LS significantly reduced the chloride ion penetration depth. Conversely, an excessive amount of lithium slag weakened this effect. The average chloride ion penetration depths of SR0, SR20, and SR40 were 9.91 mm, 7.28 mm, and 8.44 mm, respectively. Compared to the control group (SR0), the chloride ion penetration depths of SR20 and SR40 were reduced by 26.54% and 14.84%, respectively. This advantage is attributed to the optimization of the internal pore structure by LS [47,48]. The calculated chloride ion permeability coefficients for SR0, SR20, and SR40 were 0.546 × 10^−12^ m^2^/s, 0.394 × 10^−12^ m^2^/s, and 0.456 × 10^−12^ m^2^/s, respectively. By combining the trend of the strength values of S0, S20, and S40, it can be observed that the rule of the mortar’s resistance to chloride ion penetration is similar to the variation pattern of the strength values.

## 4. Conclusions

This study comprehensively analyzes the feasibility of utilizing LS as a sand substitute in mortar, examining both macro and micro perspectives. Based on experimental results, the optimal substitution rate of LS and its impact mechanism on mortar performance were determined. The key findings are summarized as follows:

(1) Due to the fine particle size and high water absorption capacity of LS, an increase in its admixture led to a gradual decrease in the fluidity of the mortar. Specifically, the fluidity of SR40 was reduced by 39.29% compared to SR0.
(2) The addition of LS first enhanced and then diminished the compressive and flexural strengths of the mortar. The initial strength enhancement is attributed to the filling effect of LS, while the subsequent decrease is caused by its inferior supporting capability compared to fine aggregates. Furthermore, the 3 d and 28 d compressive strengths of SR40 were reduced by 19.17% and 7.74%, respectively, compared to SR0.
(3) Microstructure, pore characteristics, and chloride ion permeability test results suggest that a 20% substitution rate of LS significantly optimizes the pore structure of the mortar, leading to a reduction in the chloride ion permeability coefficient. Consequently, a 20% substitution rate of LS is recommended as the optimal replacement for fine aggregates.
(4) Further research is recommended to fully understand the impact of LS as a sand substitute on the durability of mortar. It can be speculated that the optimized pore structure resulting from the incorporation of lithium slag will positively influence the durability of mortar.

## Figures and Tables

**Table 1:** The main chemical components of the raw materials.

Materials	Na_2_O	MgO	Al_2_O_3_	SiO_2_	SO_3_	K_2_O	CaO	Fe_2_O_3_	LOI
Cement	0.27	1.51	6.85	29.22	2.41	0.18	56.32	2.88	0.36
FA	2.75	0.95	14.16	53.25	2.21	2.25	9.34	7.91	7.18
LS	0.55	0.64	20.20	49.28	16.18	0.48	9.98	1.67	1.02

**Table 2:** Mix proportions of the mortar samples (kg/m^3^).

Groups	Cement	Fly Ash	Sand	LS	Water	Superplasticizer
SR0	630	90	1134	0	216	2.2
SR10	630	90	1020	114	216	2.4
SR20	630	90	907	227	216	2.8
SR30	630	90	794	340	216	3.2
SR40	630	90	680	454	216	3.6

## Data Availability

The original contributions presented in this study are included in the article. Further inquiries can be directed to the corresponding author (s).
